# Targeting CD36 With EP 80317 Reduces Remote Inflammatory Response to Hind Limb Ischemia‐Reperfusion in Mice

**DOI:** 10.1002/jbt.70057

**Published:** 2024-11-18

**Authors:** Hanan Elimam, Jade Gauvin, David N. Huynh, Liliane Ménard, Marie‐Lynn Al‐Hawat, Diala Harb, William D. Lubell, André C. Carpentier, Huy Ong, Sylvie Marleau

**Affiliations:** ^1^ Faculty of Pharmacy Université de Montréal Montréal Québec Canada; ^2^ Department of Biochemistry Faculty of Pharmacy, University of Sadat City Sadat City Egypt; ^3^ Department of Chemistry Université de Montréal Montréal Québec Canada; ^4^ Department of Medicine, Division of Endocrinology, Centre de recherche du CHUS Université de Sherbrooke Sherbrooke Québec Canada

**Keywords:** CD36, hind limb, ischemia, remote inflammation, reperfusion

## Abstract

Reperfusion of ischemic skeletal muscle triggers oxidative stress and an immediate inflammatory reaction, leading to damage of distant organs such as the lungs. The inflammatory process implicates numerous mediators, including cytokines, chemokines, and arachidonic acid metabolites. In the orchestration of the inflammatory cascade, a critical role is played by the cluster of differentiation‐36 receptor (CD36), a scavenger receptor class B protein (SR*‐*B2) which is expressed on macrophages and functions as a Toll‐like receptor coreceptor. A mouse model of hind limb ischemia‐reperfusion has been used to investigate the interplay between CD36 signaling and remote inflammation: leukocyte recruitment, regulation of the nucleotide‐binding domain leucin‐rich repeat and pyrin‐containing receptor 3 (NLRP3) inflammasome, and release of nuclear factor‐kappa B (NF‐ĸB) and arachidonic acid metabolites. Levels of reactive oxygen species, inflammatory mediators, and gene expression were measured in blood and lung tissue samples collected from anesthetized mice on which unilateral hind limb ischemia was induced by rubber band constriction for 30 min followed by reperfusion for 3 h. The CD36 modulator EP 80317, a member of the growth hormone releasing peptide 6 family, was employed as a pharmacological agent to mitigate distant lung injury following skeletal limb ischemia‐reperfusion. Targeting CD36 on monocytes/macrophages, EP 80317 abated pro‐inflammatory signaling and transcriptional activity encompassing lipid and cytokine mediators. Targeting CD36 was shown to offer promise for curtailing tissue injury following hind limb ischemia‐reperfusion.

## Graphical abstract

Proposed mechanism by which CD36 participates in generation of ROS and activation of leukocytes in the circulation and microvascular endothelium of lung tissue after reperfusion of an ischemic limb. Treatment with EP 80317, the CD36 modulator, decreased remote leukocyte recruitment, lipid mediators, cytokines, and lung tissue damage.

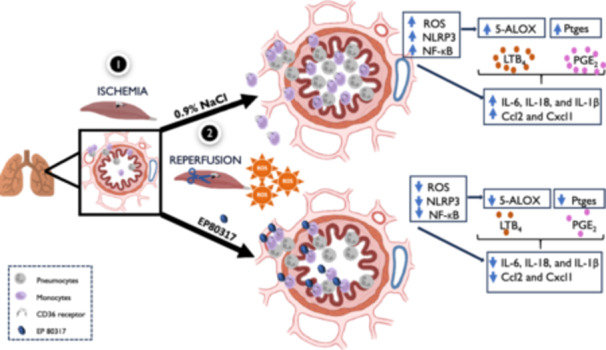

**Abbreviations**
Alox: arachidonate lipoxygenase
Alox5ap: arachidonate 5‐lipoxygenase activating protein
ARDS: acute respiratory distress syndrome
Atx: autotaxin
BHT: butylated hydroxytoluene
CCL2: chemokine C‐C motif ligand 2
CD: cluster of differentiation
Cox2: cyclooxygenase 2
Cxcl1: chemokine C‐X‐C motif ligand 1
EDTA: ethylenediaminetetraacetic acid
HTAB: hexadecyltrimethylammonium bromide
Igf‐1: insulin‐like growth factor 1
IL: interleukin
LTB_4_
: leukotriene B4
Ltb4r1: leukotriene B4 receptor 1
Ltc4s: leukotriene C4 synthase
Ly6G: lymphocyte antigen 6 complex locus G6D
MDA: malondialdehyde
MPO: myeloperoxidase
NF‐κB: nuclear factor‐kappa B
Nlrp3: nucleotide‐binding domain leucin‐rich repeat and pyrin‐containing receptor 3
NOD: Nod‐like receptor
Nox2: NADPH oxidase 2
PGE_2_
: prostaglandin E2
PMNs: polymorphonuclear neutrophils
Ptger2: prostaglandin E receptor 2 (subtype EP2)
Ptger4: prostaglandin E receptor 4 (subtype EP4)
Ptges: prostaglandin E synthase
ROS: reactive oxygen species
Siglecf: sialic acid binding Ig‐like lectin F
SR‐B2: scavenger receptor B2
TBARS: thiobarbituric acid reactive substance
TLR: toll‐like receptor
TMB: 3,3′,5,5′‐tetramethylbenzidine
TNF‐α: tumor necrosis factor alpha

## 1 Introduction

Reperfusion of skeletal ischemic tissue is crucial for functional recovery but leads paradoxically to remote organ injury [1]. For example, respiratory complications and lung damage have been correlated with the duration of ischemia preceding reperfusion [2]. Within the lungs, alveolar macrophages and monocyte‐derived lung macrophages play vital roles in innate defense mechanisms and respond to the sterile inflammatory response to ischemia and reperfusion [3]. In response to infection and injury, circulating polymorphonuclear neutrophils (PMNs) and monocytes are recruited to the lungs [2]. In the lungs, alveolar and interstitial macrophages play a crucial role in innate defense against infections and contribute to resolving sterile inflammation [4]. Reactive oxygen species (ROS) are generated and released in early events after hind limb ischemia and reperfusion and promote subsequent biosynthesis of arachidonic acid metabolites [5, 6]. Skeletal muscle tissue injury elicits release of inflammatory cytokines and systemic inflammation [7, 8, 9]. In rodent and rabbit models exposed to hind limb ischemic insult followed by reperfusion, lung damage has been shown to be due in part to activated PMNs, which are mainly trapped within lung capillaries [10, 11, 12, 13, 14]. In remote lung damage following hind limb ischemia and reperfusion, the causative and amplifying contributions remain incompletely characterized for PMNs, monocytes, and macrophages as sources of inflammatory mediators.

The cluster of differentiation 36 receptor [CD36, scavenger receptor B2 (SR‐B2)] is a Toll‐like receptor (TLR) coreceptor which regulates activation of the CD36/TLR heterodimer complex and consequent transcription of pro‐inflammatory cytokines, production of nitric oxide, and generation of ROS [15]. Situated on the cellular surfaces of monocytes and macrophages, the CD36/TLR heterodimer complex responds to endogenous ligands and plays pivotal roles in inflammation and innate immunity [16]. As coreceptor, CD36 is linked to the immune response and physio‐pathological conditions which share inflammation as a common feature, including atherosclerosis, atherothrombosis, diabetes, stroke, Alzheimer's disease, angiogenesis, and cancer [17]. In the lungs, CD36 is expressed on the surfaces of alveolar macrophages, type 2 pneumocytes, and interstitial macrophages, as well as blood‐derived monocytes, which are recruited during inflammation [18, 19]. The severity of tissue damage has been shown to be mediated by CD36 in brain ischemia [20] and myocardial ischemia‐reperfusion [21, 22, 23], as well as in acute lung injury induced by H_2_O_2_ [24] and lipopolysaccharide [25]. The latter is associated with M1 polarization and nuclear factor‐kappa B (NF‐κB) activation [25]. In isolated mononuclear phagocytes, CD36 plays a pro‐inflammatory role by activating NF‐κB and the Nod‐like receptors (NLR) family pyrin domain containing 3 (NLRP3) inflammasome [15].

Hypothesizing that activation of CD36 on monocytes and macrophages in the lungs could potentiate inflammation, the present study employs genetic and pharmacological methods to investigate the role of the coreceptor in regulating leukocyte recruitment to inflammatory sites in an acute inflammatory murine model of hind limb ischemia and reperfusion. The ligand EP 80317 has previously been shown to modulate inflammatory responses in a CD36‐dependent manner [21, 26]. Employing EP 80317 to modulate CD36 signaling, the relevance of the latter in exacerbating the inflammatory response has been indicated by measuring decreased production of cytokines, ROS, and arachidonic acid metabolites.

## 2 Materials and Methods

### 2.1 Animals

CD36‐deficient (CD36^−/−^) mice and wild‐type littermate controls on a C57Bl/6 J background were obtained from Jackson Laboratories (Bar Harbor, Maine, USA) and bred following established protocols. The mice were housed and cared for in local animal facilities, maintained on a 12:12‐h light/dark cycle, and provided with standard conditions, including ad libitum access to food and water. Thirty‐two wildtype (CD36^+/+^) and twelve CD36^−/−^ male mice aged between 12 and 14 weeks were equally divided into two groups (control and EP 80317) and utilized for all experimental procedures. The Institutional Animal Ethics Committee approved all experimental protocols, ensuring compliance with guidelines outlined by the Canadian Council on Animal Care and the US National Institute of Health for the ethical treatment and use of laboratory animals.

### 2.2 Drug and Experimental Protocol

The drug EP 80317 (H‐Haic–D‐2MeTrp–d‐Lys–Trp–d‐Phe–Lys‐NH_2_) was obtained from Europeptides (Argenteuil, France) and reconstituted in sterile 0.9% NaCl (Baxter Corporation, Toronto, Ontario, Canada) before parenteral administration. The control group was treated with sterile 0.9% NaCl. For 14 days, CD36^+/+^ and CD36^‐/‐^ mice were subcutaneously injected daily with either EP 80317 (300 μg/kg) or 0.9% NaCl, excluding the day of experimentation. Following anesthesia with isoflurane, mice underwent a 30‐min ischemia of the right hind limb induced by applying a rubber band (black latex o‐ring, Miltex, York, Germany) above the greater trochanter using a McGivney hemorrhoidal ligator (Miltex, York, Germany). After 3 h of hind limb reperfusion, mice were euthanized via isoflurane overdose and exsanguination ( ~ 1000 µL in heparinized tubes) from the jugular vein. Lung tissues were collected and stored at –80°C until further analysis.

### 2.3 Histology

Lung tissues were fixed in formalin, sectioned into 5 µm‐thick slices, processed, and subsequently stained with hematoxylin and eosin by McGill University Health Centre's histopathology platform. Images were captured using a 40X objective using a NanoZoomer 2.0‐HT digital scanner and NDP view2 software (Hamamatsu Photonics, Shizuoka, Japan). Cells were counted manually using Adobe Photoshop CS3 software (San José, CA, United States) by two individuals who were blinded from knowledge of prior treatments.

### 2.4 Myeloperoxidase Assay in Lung Homogenates

A standard curve was constructed to determine the number of PMNs in tissue. PMNs were harvested from the mice peritoneal cavity 16 h after an intraperitoneal injection of 2 mL of 5% casein (Sigma‐Aldrich, St Louis, MO, USA), euthanasia was performed, and samples were collected and purified by positive selection (> 98% PMN) using an anti‐lymphocyte antigen 6 complex locus G6D (anti‐Ly6G) magnetic microbead kit (Miltenyi Biotec, Auburn, CA), according to the manufacturer's instructions. Hypotonic lysis of red blood cells was performed by resuspension of the cells in 0.2% NaCl for 20 s, followed by the addition of an equal volume of 1.6% NaCl. Aliquots of neutrophils (10^6^ cells/mL) were stored at –80°C in acetate buffer (100 mM), pH 6, supplemented with 1% hexadecyltrimethylammonium bromide (HTAB) (Sigma‐Aldrich, St Louis, MO, USA) and 20 mM ethylenediaminetetraacetic acid (EDTA) (Sigma‐Aldrich, St Louis, MO, USA). Thawed aliquots were homogenized, incubated at 65°C for 2 h, centrifuged at 2000 g for 10 min, and the supernatants were used to construct the MPO standard curve. Lung tissue MPO was assayed as previously described, with slight modifications [27]. The lung tissue was homogenized in 1 mL PBS (Gibco Life Technologies, Grand Island, NY, USA), followed by centrifugation. The resulting pellets were suspended in 1 mL acetate buffer with 1% HTAB and 20 mM EDTA before undergoing another round of homogenization. Lung homogenates were heated to 65°C for 2 h, subjected to 3 freeze–thaw cycles, and then centrifuged at 2000 g for 10 min. Lung tissue MPO was assayed by incubating supernatants with 3.2 mM of 3,3′,5,5′‐tetramethylbenzidine (TMB, Sigma‐Aldrich, St Louis, MO, USA) and 1.0 mM of H_2_O_2_ (Sigma‐Aldrich, St Louis, MO, USA) for 5 min at 37°C. The reaction was stopped by the addition of 100 µL of 0.2 M sodium acetate (pH 3). Equivalent numbers of PMNs per tissue homogenate were calculated from the standard curve and normalized per g.

### 2.5 Real Time‐Quantitative Polymerase Chain Reaction (RT‐qPCR) in Lung Tissue

Total mRNA was extracted from lung tissue with the Ribozol RNA Extraction Reagent (VWR International, Radnor, PA, USA) with the PureLink^TM^ RNA Micro‐Kit (Invitrogen, Waltham, MA, USA), as described previously [28]. Relative mRNA expression levels of genes were determined using the comparative CT (2^‐ΔΔCt^) method, and results were normalized to the mean of 5 internal controls, *βactin*, *Gapdh*, *Hprt*, *Rpl13a*, and *Ywhaz*. The murine primer sequences are detailed in Table 1.

**Table 1:** qPCR murine primer sequences.

Gene	Primer	Product length (bp)	NCBI gGene ID
*Βactin*	Forward CAGCAAGCAGGAGTACGATGA	93	11461
	Reverse GAAAGGGTGTAAAACGCAGCTC		
*Alox5*	Forward GTCCTGAGGGATGGACGTGCAAAAT	89	11689
	Reverse TGCCGTGCCTCCAGTTCTTTACG		
*Alox5ap*	Forward GGACTCTTGCCTTTGAGCGGGT	139	11690
	Reverse TGCCGAAGATGTAGCCAGGGGT		
*Alox12*	Forward AGTGCGTTTGTGGCTGGTTGGG	90	11684
	Reverse AAGTCAAACTCCTCCTCCTTGCCCC		
*Alox15*	Forward TGGGGCAACTGGAAGGATGGCA	138	11687
	Reverse AACGGTGTCCATTGTCCCCAGAAC		
*Atx*	Forward GACCCTAAAGCCATTATTGCTAA	81	18606
	Reverse GGGAAGGTGCTGTTTCATGT		
*Ccl2*	Forward TCATGCTTCTGGGCCTGCTGTTCA	101	20296
	Reverse GAATGAGTAGCAGCAGGTGAGTGGG		
*Cd11c*	Forward CAGAGCCAGAACTTCCCAACTGCAC	87	16411
	Reverse GATGCTACCCGAGCCATCAATCAGG		
*Cox2*	Forward GGACTGGGCCATGGAGTGGACTTAAA	124	19225
	Reverse GGGGATACACCTCTCCACCAATGACC		
*Cxcl1*	Forward CACCCAAACCGAAGTCATAGCCACA	125	14825
	Reverse TCTTTCTCCGTTACTTGGGGACACCT		
*Ptger2*	Forward TTGCCATACTTAGGCCACCG	153	19217
	Reverse CGCATCCTCACAACTGTCCA		
*Ptger3*	Forward ATTGCAGTTCGCCTGGCTTCGC	127	19218
	Reverse AGGTGGAGCTGGAAGCATAGTTGGT		
*Ptger4*	Forward GTGCTCATCTGCTCCATTCCGCT	108	19219
	Reverse CCTGATGGCCTGCAAATCTGGGTT		
*Gapdh*	Forward TCGGTGTGAACGGATTTGGCCG	147	14433
	Reverse TGCCGTGAGTGGAGTCATACTGGA		
*Hprt*	Forward TCCTCCTCAGACCGCTTTTTGCC	80	15452
	Reverse CATCGCTAATCACGACGCTGGGA		
*Igf1*	Forward GCTTTTACTTCAACAAGCCCACAGGC	150	16000
	Reverse AGCGGGCTGCTTTTGTAGGCT		
*Il1β*	Forward GGACCCCAAAAGATGAAGGGCTGC	146	16176
	Reverse TGCCACAGCTTCTCCACAGCCA		
*Il6*	Forward CTCTGGAGCCCACCAAGAACGA	84	16193
	Reverse AAGGCAACTGGATGGAAGTCTCTTGC		
*Il18*	Forward TGCCAGTGAACCCCAGACCAGA	86	16173
	Reverse CCTTCACAGAGAGGGTCACAGCCA		
*Ltb4r1*	Forward TGGCTGTGTTGCTCACTGCTCC Reverse ACAGGCGGCAACCCATCTCTCT	84	16995
*Ltc4s*	Forward CCCTGTGCGGACTGTTCTACCTGT	93	17001
	Reverse GCAGGAGCATCTGGAGCCATCTGA		
*Nfκb1*	Forward GGATTTGCTGAGGGTTGGGGCT	131	18033
	Reverse GGGGCGCTGCTTTTCTGCTCTT		
*Nfκb2*	Forward AGCAGGAGGCCAAGGAGCTGAA	114	18034
	Reverse TCACAGGCTTCAGGGGCAAGGA		
*Nlrp3*	Forward AAGGACCAGCCAGAGTGGAATGACA	108	216799
	Reverse GCGGGAGAGATATCCCAGCAAACCC		
*Nox2*	Forward TGCTGGAGACCCAGATGCAGGAA	102	13058
	Reverse GCACAGCAAAGTGATTGGCCTGAGAT		
*Ptges*	Forward CAAGATGTACGCGGTGGCTGTCA	90	64292
	Reverse CCTCCACGTTTCAGCGCATCCT		
*RelA*	Forward ATCGAACAGCCGAAGCAACGGG	112	19697
	Reverse TGGTGGGGTGTGTCTTGGTGGT		
*RelB*	Forward GGTTCCAGTGACCTCTCTTCCCTGT	90	19698
	Reverse CAGGCCAAAGCCGTTCTCCTTAATGT		
*Rpl13a*	Forward GAAGCAGATCTTGAGGTTACGGA	131	22121
	Reverse GCAGGCATGAGGCAAACAGT		
*Siglecf*	Forward CCAAAGGTCTCACAGGCAGGCAA	100	233186
	Reverse AGAGGCAGACAGCAAGCAAGCC		
*Tnf*	Forward TGTAGCCCACGTCGTAGCAAACCA	149	21926
	Reverse CCTGGGAGTAGACAAGGTACAACCCA		
*Ywhaz*	Forward TCCCCAATGCTTCGCAACCAGAA	126	22631
	Reverse CTTGCTGTGACTGGTCCACAATTCCT		

### 2.6 Cytokine and Chemokine Levels in Lung Homogenates

The lung homogenates underwent analysis using commercial ELISA kits (eBioscience, Waltham, MA, USA) specific for IL‐1β (#88‐7013), following the manufacturer's guidelines.

### 2.7 PGE_2_ and LTB_4_ Levels in Lung Homogenates

Commercial ELISA kits (R&D System Inc., Minneapolis, USA) were used to measure PGE_2_ (# KGE004B) and LTB_4_ (# KGE006B) levels in lung homogenates using a competitive binding assay according to the manufacturer's instructions.

### 2.8 Malondialdehyde Plasma Levels

Malondialdehyde (MDA) which was generated from the breakdown of primary and secondary lipid peroxidation products, was quantified using the thiobarbituric acid reactive substance (TBARS) assay (Sigma‐Aldrich, St Louis, MO, USA). Plasma samples (25 μL) were mixed with PBS (475 μL) and incubated with 30 μL of butylated hydroxytoluene (BHT) and 1 mL of TBARS reagents for 60 min in glass tubes heated to 95°C in a heating block. After cooling, the samples were centrifuged at 1100 g for 10 min at 4°C, and the absorbances were measured at 532 nm. MDA concentrations were determined using the equation: MDA = Absorbance/1.56 × 10^5^ (mole/L) as the molar absorbance coefficient (Kheradmand, Alirezaei, Asadian, Rafiei Alavi, & Joorabi, 2009).

### 2.9 Statistical Analysis

Data were analyzed using GraphPad Prism version 8.4.3 (San Diego, CA, USA) and expressed as mean ± SEM. To determine significant differences among groups in CD36^+/+^ and CD36^‐/‐^ mice, individual comparisons were made between groups using an unpaired *t* test with Welch's correction or the Mann–Whitney test. Statistical significance was considered at a *p* value < 0.05.

## 3 Results

### 3.1 EP 80317 Attenuates Plasma and Lung ROS and Inflammatory Mediators Following Hind Limb Ischemia and Reperfusion in a CD36‐dependent Manner

**Figure 1:**
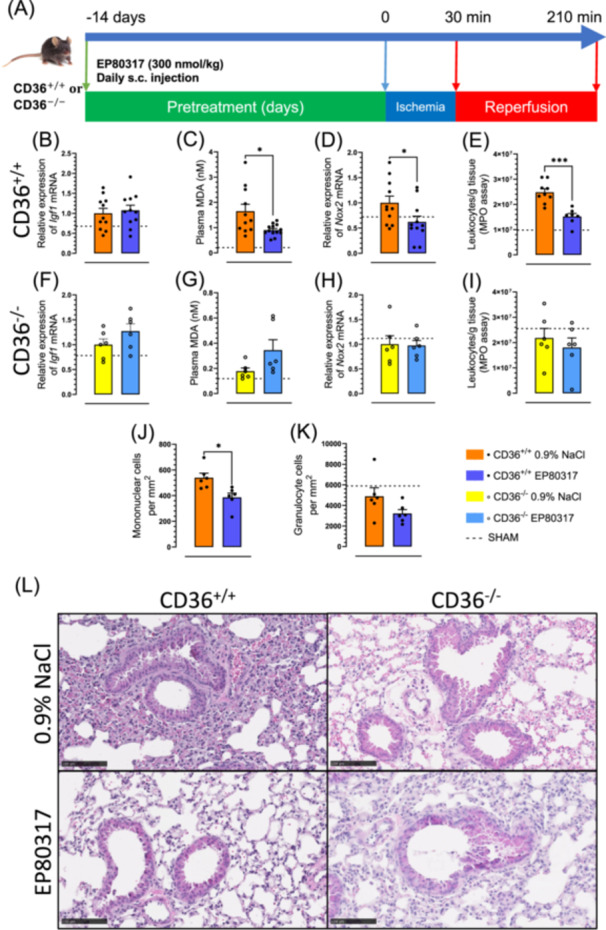
EP 80317 reduces systemic and lung homogenate ROS and inflammatory mediators. (A) Study design. (B) Bar graphs and dot plots represent the relative expression of *Igf1* mRNA in lung tissue of CD36^+/+^ and (F) CD36^‐/‐^ mice. (C) Mean plasma MDA levels of CD36^+/+^ and (G) CD36^‐/‐^ mice, expressed as bar graphs and dot plots. (D) Bar graphs and dot plots represent the relative expression of *Nox2* mRNA in lung tissue of CD36^+/+^ and (H) CD36^‐/‐^ mice. (E) Bar graphs and dot plots of the total leucocytes recruitment in lung tissue by MPO assay of CD36^+/+^ and (I) CD36^‐/‐^ mice. (J) Bar graphs and dot plots represent the mononuclear cells count per mm^2^ of photomicrographs of lung tissue of CD36^+/+^ and (K) CD36^‐/‐^. (L) Representative photomicrographs of lungs after staining with hematoxylin‐eosin (scale bar: 100 μm). Data are mean ± SEM. *p < 0.05 and ***p < 0.001, as assessed by an unpaired t test. *n* = 6–11 per group for CD36^+/+^ and *n* = 6 per group for CD36^‐/‐^.

Wildtype (CD36^+/+^) and CD36‐deficient (CD36^‐/‐^) mice were pretreated daily with EP 80317 for 14 days before being subjected to a 30 min unilateral hind limb ischemia followed by 180 min reperfusion (I30/R180) (Figure 1A). EP 80317, identified previously as a nongrowth hormone secretagogue [29], did not alter the relative expression of *Igf1* mRNA levels in lung tissue of CD36^+/+^ and CD36^‐/‐^ mice (Figure 1B,F). Mice treated with EP 80317 showed reduced ROS levels both systemically (Figure 1C,G) and remotely in lungs (Figure 1D,H) in a CD36‐dependent manner following hind limb ischemia and reperfusion. Using the MPO assay, total leukocyte recruitment into the lungs was assessed and shown to be reduced by 39% (*p* < 0.001) after pretreatment with EP 80317 compared to 0.9% NaCl vehicle in CD36^+/+^ but not CD36^‐/‐^ mice (Figure 1E,I). Manual microscopic analysis revealed a 28% decrease of mononuclear phagocyte cells (*p* < 0.05) (Figure 1J) without significant effect on granulocytes (Figure 1K), validating the effect of EP 80317 on CD36^+/+^ mice. Alveolar macrophage counts were unaltered in EP 80317‐treated versus vehicle‐treated mice (449 ± 32 vs. 495 ± 17 cells per mm^2^). Preserved alveolar structure, less vascular congestion, and diminished leukocyte accumulation, were observed in the photomicrographs of right lung sections from EP 80317‐treated CD36^+/+^ mice compared to those from vehicle‐treated CD36^+/+^ and CD36^‐/‐^ counterparts (Figure 1L).

**Figure 2:**
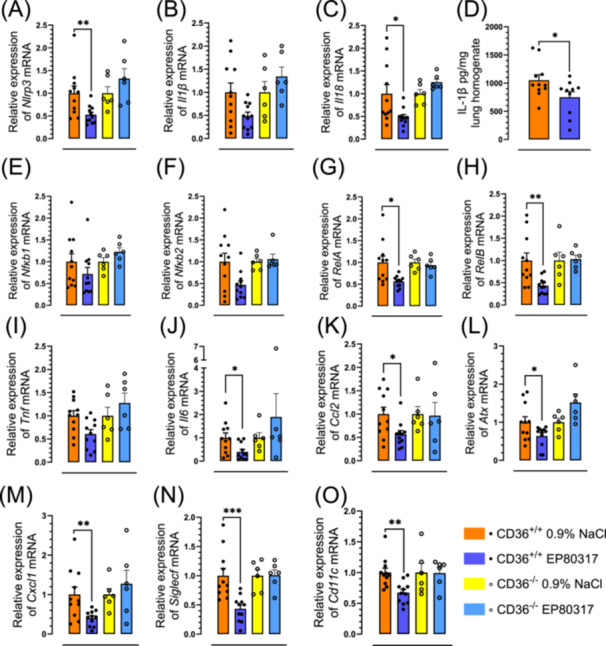
EP 80317 reduces lung NLRP3 inflammasome and pro‐inflammatory cytokines and chemokines. Bar graphs and dot plots represent the relative expression of (A) *Nlrp3*, (B) *Il1β*, and (C) *Il18* mRNA in lung tissue of CD36^+/+^ and CD36^‐/‐^ mice. (D) Mean IL‐1β levels in lung homogenates of CD36^+/+^ mice, expressed as bar graph and dot plot. Bar graphs and dot plots represent the relative expression of (E) *Nfκb1*, (F) *Nfκb2*, (G) *RelA*, (H) *RelB*, (I) *Tnf*, (J) *Il6*, (K) *Ccl2*, (L) *Atx*, (M) *Cxcl1*, (N) *Siglecf*, and (O) *Cd11c* mRNA in lung tissue of CD36^+/+^ and CD36^‐/‐^. Data are mean ± SEM. *p < 0.05, **p < 0.01, and ***p < 0.001, as assessed by an unpaired t test. *n* = 11 for CD36^+/+^ and *n* = 6 for CD36^‐/‐^.

Examination of mRNA extracted from the lungs of treated mice demonstrated that EP 80317 reduced *Nlrp3*, *Il1β*, *Il18* gene expression and IL‐1β protein levels in a CD36‐dependent manner (Figure 2A–D). Moreover, EP 80317 diminished mRNA levels of nuclear factor‐kappa B (*Nfκb*) family members (Figure 2E–H), primarily the noncanonical *RelA* and *RelB* in a CD36‐dependent manner. Concurrent with reduced NF‐κB signaling, EP 80317 lowered gene expression of pro‐inflammatory cytokines and chemokines: e.g., tumor necrosis factor (*Tnf*) (*p* < 0.05) (Figure 2I), *Il6* (*p* < 0.05) (Figure 2J), chemokine C‐C motif ligand 2 (*Ccl2*) (*p* < 0.05) (Figure 2K), and autotaxin (*Atx*) (*p* < 0.05) (Figure 2L). Furthermore, EP 80317 caused a reduction in mRNA expression for the chemokine (C‐X‐C motif) ligand 1 (*Cxcl1*) (Figure 2M) and for markers of granulocyte presence such as sialic acid binding Ig‐like lectin F (*Siglecf*) and *Cd11c* (Figure 2N–O). In contrast, mRNA levels of these biomarkers were unchanged in EP 80317‐treated CD36^‐/‐^ mice and vehicle treated CD36^+/+^ mice (Figure 2A–O).

### 3.2 EP 80317 Decreases Arachidonic Acid Metabolites in Hind Limb Ischemia and Reperfusion

**Figure 3:**
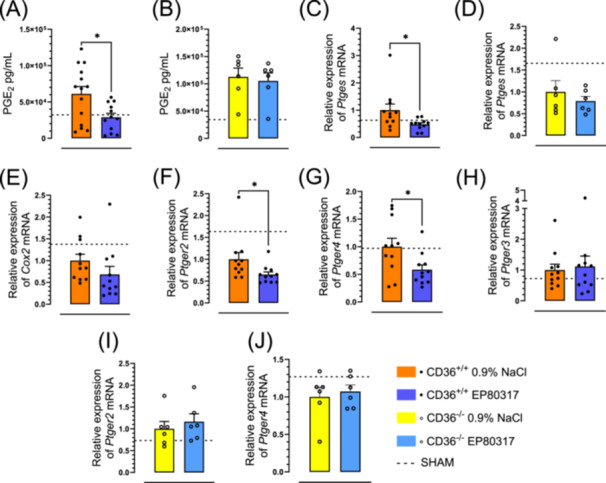
EP 80317 decreases arachidonic acid metabolites‐PGE_2_. PGE_2_ levels in lung homogenates of (A) CD36^+/+^ and (B) CD36^‐/‐^ mice, represented as bar graphs. Bar graphs and dot plots represent the relative expression of *Ptges* mRNA in lung tissue of (C) CD36^+/+^ and (D) CD36^‐/‐^ mice. (E) Bar graph and dot plot represent the relative expression of *Cox2* mRNA in lung tissue of CD36^+/+^. Relative expression of (F) *Ptger2*, (G) *Ptger4*, and (H) *Ptger3* mRNA in lung tissue of CD36^+/+^ mice, expressed as bar graphs and dot plots. Bar graphs and dot plots represent the relative expression of (I) *Ptger2* and (J) *Ptger4* mRNA in lung tissue of CD36^‐/‐^ mice. Data are mean ± SEM. *p < 0.05, as assessed by an unpaired *t* test. *n* = 14 for CD36^+/+^ and *n* = 6 for CD36^‐/‐^.

In lung homogenates of EP 80317‐treated CD36^+/+^ mice subjected to ischemia and reperfusion, the levels of PGE_2_ were decreased by 53% (*p* < 0.05) from 6.1 ± 1.0 × 10^4^ to 2.9 ± 0.6 × 10^4 ^pg/mL compared to vehicle‐treated mice (Figure 3A). Pretreatment with EP 80317 caused no change on PGE_2_ levels in CD36^‐/‐^ mice (Figure 3B). After EP 80317 treatment, prostaglandin E synthase (*Ptges*) mRNA levels were reduced in CD36^+/+^ but not in CD36^‐/‐^ mice compared to vehicle (Figure 3C,D). No significant change was observed in cyclooxygenase 2 (*Cox2*) mRNA levels between groups (Figure 3E). The expression levels of the prostaglandin E receptors *Ptger2* (Figure 3F) and *Ptger4* (Figure 3G) but not *Ptger3* (Figure 3H) were reduced by treatment of CD36^+/+^ mice with EP 80317, which had no effect on CD36^‐/‐^ mice (Figure 3I,J).

**Figure 4:**
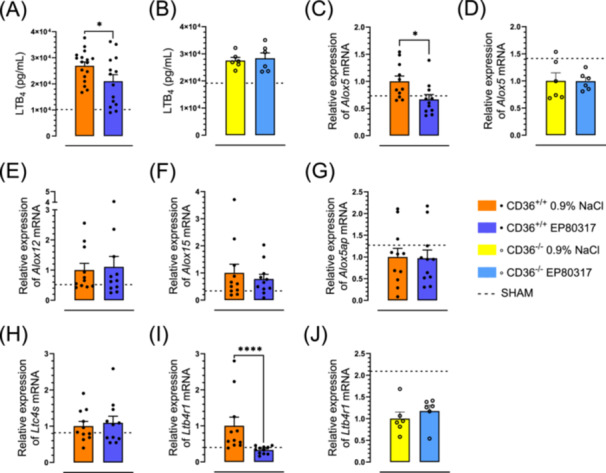
EP 80317 decreases arachidonic acid metabolites‐LTB_4_. (A) Bar graph and dot plot represent the LTB_4_ levels in lung homogenates of CD36^+/+^ and (B) CD36^‐/‐^ mice. (C) Relative expression of *Alox5* mRNA in lung tissue of CD36^+/+^ and (D) CD36^‐/‐^ mice. Bar graph and dot plot represent the relative expression of (E) *Alox12*, (F) *Alox15* and (G) *Alox5ap* mRNA in lung tissue of CD36^+/+^ mice. (H) Bar graph and dot plot of the relative expression of *Ltc4s* mRNA in lung tissue of CD36^+/+^. (I) Bar graph and dot plot represent the relative expression of *Ltb4r1* mRNA in lung tissue of CD36^+/+^ and (J) CD36^‐/‐^ mice. Data are mean ± SEM. *p < 0.05 and ***p < 0.001, as assessed by an unpaired *t* test. *n* = 14 for CD36^+/+^ and *n* = 6 for CD36^‐/‐^.

LTB_4_ levels in lung homogenates were increased by 2.7‐fold in CD36^+/+^ mice subjected to ischemia and reperfusion (Figure 4A) and compared to vehicle‐treated mice, were reduced by 22% (*p* < 0.05) from 2.7 ± 0.1 × 10^4^ to 2.1 ± 0.3 × 10^4 ^pg/mL upon treatment with EP 80317, which did not affect LTB_4_ levels in CD36^‐/‐^ mice (Figure 4B). Arachidonate 5‐lipoxygenase (*Alox5*) mRNA levels were reduced by 1.6‐fold in EP 80317‐treated CD36^+/+^ mice, back to baseline levels of sham‐operated mice (Figure 4C), but no effect of the ligand was observed in CD36^‐/‐^ mice (Figure 4D). In contrast, EP 80317 pretreatment had no effect on the mRNA levels of *Alox12* (Figure 4E), *Alox15* (Figure 4F), arachidonate 5‐lipoxygenase activating protein (*Alox5ap*) (Figure 4G), nor leukotriene C4 synthase (*Ltc4s*) (Figure 4H). Finally, leukotriene B4 receptor 1 (*Ltb4r1*) mRNA expression was reduced 3.3‐fold in CD36^+/+^ mice pretreated with EP 80317 and subjected to ischemia and reperfusion (Figure 4I), but no effect was observed in the CD36^‐/‐^ counterpart (Figure 4J).

A mechanism is proposed by which CD36 participates in the generation of ROS and activation of leukocytes in the circulation and microvascular endothelium of lung tissue after reperfusion of an ischemic limb. Treatment with EP 80317 attenuates the cascade of cytokines, chemokines, and arachidonic acid metabolites by mitigating CD36 signaling. Consequently, the CD36 modulator decreased remote leukocyte recruitment, lipid mediators, cytokines, and lung tissue damage.

## 4 Discussion

Remote injury following the reperfusion of an ischemic limb is known to affect well‐vascularized organs, such as the lungs, liver, and intestines. Consequences include organ injury and acute respiratory distress syndrome (ARDS). The principal finding of this study is that the scavenger receptor CD36 plays a critical role in these events by amplifying expression of key inflammatory mediators and recruiting leukocytes to vascularized tissue.

In skeletal muscle reperfusion, ROS play a well‐documented role in the release of pro‐inflammatory mediators, such as prostaglandins, thromboxane A2, and leukotriene B4, which mutually prime formation and activate leukocytes within the pulmonary circulation [10, 30, 31, 32]. Accordingly, pulmonary *Nox2* expression and plasma MDA levels were decreased in EP 80317‐treated mice in a CD36‐dependent manner. Expression of *Nox2* and *p40phox* were previously shown to decrease in atherosclerotic vascular tissue of apolipoprotein E (apoE)‐null mice treated with EP 80317 [33]. Moreover, a decrease in oxidative stress was observed in the left ventricle of mice that were treated with a selective azapeptide CD36 modulator before transient myocardial ischemia [23]. The systemic inflammatory response triggered by the reperfusion of the ischemic limb leads to remote organ injury, with greatest severity to the lungs [14, 34]. Activated vascular leukocytes are suggested to contribute to organ damage by releasing TNF‐α and IL‐1β, which upregulate cell adhesion molecules [7, 8, 35]. Injury of the lungs after limb reperfusion is associated with extensive infiltration of mononuclear cells as well as recruitment of PMNs, which mediate inflammatory responses [36, 37]. In the present study, lung tissue MPO and microscopic analysis of leukocyte counts indicated mononuclear phagocyte accumulation, which was curtailed by the CD36 modulator EP 80317.

Animal studies have highlighted the role of CD36 in cardiovascular diseases, particularly atherosclerosis [38] and atherothrombosis [39]. Previously, CD36‐selective ligands have reduced macrophage accumulation in aortic lesions, diminished foam cell formation, and mitigated atherosclerosis progression in apoE‐null mice [33]. Consistent with its role in chronic inflammatory diseases such as atherosclerosis, CD36 modulation was associated with reduced systemic inflammation and increased differentiation of vascular macrophages towards an M2 anti‐inflammatory phenotype [28, 29, 40]. The findings from a systematic review and meta‐analysis in humans demonstrated a significant association between the CD36 rs1761667 polymorphism and cardiometabolic risk factors, including circulating triglycerides, HDL cholesterol, and fasting blood glucose levels [41]. In a model of subretinal inflammation elicited by photo‐oxidative stress, the modulatory activity of the CD36 ligand azapeptide MPE‐298 reduced mononuclear phagocyte accumulation and production of inflammatory mediators with a change in cellular energy metabolism towards oxidative phosphorylation [15] and reduced mitochondrial stress in retinal pigment epithelium [42]. Azapeptide MPE‐298 has exhibited vascular protective effects and contributed to lesion regression in models of atherosclerosis [40]. In chronic obstructive pulmonary disease (COPD), CD36 has been shown to play a role in the progression of emphysema, particularly in the context of elastase‐induced disease advancement [43]. The role of CD36 expression in microvascular endothelial cells and circulating monocytes has now been studied in an acute inflammation model featuring hind limb ischemia and reperfusion.

The NLRP3 inflammasome is activated by CD36 signaling in response to TLR‐2 activation by lipopeptide and ROS [15]. Reduced levels of IL‐1β and decreased expression of *Nlrp3* and *Il18* mRNA were consistently found in lung homogenates from animals treated with EP 80317 before reperfusion compared to vehicle‐treated and CD36‐null counterparts. In addition to inhibiting the inflammasome NLRP3, EP 80317 caused a reduction in the expression of cytokines, chemokines, and inflammatory markers: *Tnf*, *Il*6, *Ccl2*, *Cxcl1*, *Atx*, *Siglecf*, and *CD11c*. The recruitment and localization of leukocytes in the lungs are significantly impacted by CD36 and likely involve both mononuclear phagocytes and polymorphonuclear leukocytes.

In mice subjected to acute inflammation caused by scorpion envenomation, treatment with EP 80317 effectively decreased leukocyte accumulation, prostaglandin E2, and IL‐1β levels remotely in bronchoalveolar fluid [44]. The latter activity of EP 80317 was attributed to effects that decreased NF‐κB phosphorylation and, in part, caused reductions of AMPc and PKA activation elicited by LTB_4_ and consequently inhibited NF‐κB activation. In mice subjected to myocardial ischemia‐reperfusion, the CD36 ligand azapeptide CP‐3(iv), increased circulating adiponectin levels, epididymal fat adiponectin gene expression, and transcriptional regulators (Pparg, Cebpb, Sirt1) after 6 h of reperfusion. Additionally, azapeptide CP‐3(iv) reduced myocardial oxidative stress and apoptosis [23]. In the context of lung inflammation and disease [24, 25, 43], a CD36 ligand reduced the cytokine storm elicited in experimental SARS‐Cov‐2 infected mice [45]. To our knowledge, no study has reported a link between remotely induced lung injury and CD36 expressed by alveolar or incoming mononuclear cells.

Our findings align with previous observations indicating what caused a decrease in the expression of *Nfκb1* and *Nfκb2* in lung tissue at the mRNA level. In the context of skeletal muscle limb ischemia and reperfusion, COX2 inhibition has been shown to prevent remote pulmonary dysfunction and increase permeability [10, 46]. In the current study, levels of PGE_2_ were reduced in lung homogenates from EP 80317‐treated mice, but not in CD36‐null counterparts. This reduction was associated with decreased expression of PGE_2_ synthase and reduced mRNA levels for the *Ptger2* and *Ptger4* receptors. In contrast, EP 80317 had no significant effect on *Cox2* nor *Ptger3* mRNA levels. Among the EP receptors, EP2 and EP4 bind PGE_2_ with high affinity and promote inflammation, consequently causing cytokine release and immune cell recruitment [47]. A previous study showed that PGE_2_‐mediated increase in IL‐1β is dependent on EP2 and EP4 signaling using a murine bone marrow transplant model [47, 48].

In systemic inflammation caused by hind limb ischemia and reperfusion, PGE_2_ has anti‐inflammatory properties and decreases cytokine secretion, but has also been linked with detrimental outcomes in the lungs such as heightened vascular permeability and plasma protein extravasation [49]. The anti‐inflammatory impact of PGE_2_, which curbs cytokine secretion and lung macrophage invasion, is facilitated through Ptger2 and Ptger4 signaling, underscoring an intricate role of PGE_2_ in lung inflammation secondary to systemic inflammation, which warrants further investigation. Arachidonic acid metabolism following hind limb ischemia and reperfusion leads to activated endothelial cell and leukocyte production of the powerful chemoattractant LTB_4_, which is released both systemically and locally in lung tissue [8, 36, 50]. In the context of the hind limb ischemia and reperfusion model, the inflammatory effects of CD36 were alleviated by EP 80317 treatment, which decreased LTB_4_ and IL‐1β levels as well as reduced *Alox5, Ltb4r1*, and *Nlrp3* inflammasome mRNA expression in lung tissue but caused no alterations in the mRNA levels of *Alox12*, *Alox15*, *Alox5ap* nor *Ltc4s*. Expression of the *Ltb4r1* receptor leads to intracellular signaling cascades through G proteins, affecting various cellular responses such as chemotaxis of immune cells, cytokine production, and oxidative burst [51]. In the present study, the reduction of levels of LTB_4_ and *Ltb4r1* leads to inhibition of the inflammatory response. An inflammatory CD36‐LTB_4_ pathway is suggested in this model. More translational studies are needed to confirm the role of CD36 in remote organ injury. In a sequence of events leading to remote injury upon reperfusion of the ischemic hind limb, the reintroduction of oxygen may lead to the generation of ROS, triggering an acute inflammatory response in vascular endothelial cells and circulating leukocytes. The latter accumulate in well‐perfused tissues, become trapped, and migrate into the lung, upregulating NF‐κB‐dependent cytokine gene expression and NLRP3 inflammasome activation. Expressed by pulmonary macrophages, CD36 is an innate immune receptor that regulates the expression of pro‐inflammatory genes, such as *Ptges* and *Alox5*, and receptors on leukocytes. Treatment with a CD36 modulating ligand reduces lung leukocytosis as well as systemic and lung inflammatory mediator levels. Moreover, CD36‐deficient mice exhibit a similar pattern of events. Future research will explore the initial signaling molecules that regulate the formation of ROS, activate the NLRP3 inflammasome, and trigger NF‐ĸB inflammatory pathways, dissecting the sequential and concomitant pathways occurring in the inflammatory response. Targeting CD36 in hind limb ischemia and reperfusion could serve as a promising upstream pharmacological target.

## Author Contributions

**Hanan Elimam:** conceptualization, methodology, investigation, formal analysis, writing original draft, writing–review and editing. **Jade Gauvin:** formal analysis, writing original draft, writing–review and editing. **David N. Huynh:** methodology, investigation, formal analysis, writing–review and editing. **Liliane Ménard:** methodology, investigation, formal analysis, writing–review and editing. **Marie‐Lynn Al‐Hawat:** writing original draft, writing–review and editing. **Diala Harb:** writing–review and editing. **William D. Lubell:** resources, writing–review and editing. **André C. Carpentier:** funding acquisition, writing–review and editing. **Huy Ong:** funding acquisition, writing–review and editing. **Sylvie Marleau:** conceptualization, funding acquisition, writing original draft, writing–review and editing.

## Conflicts of Interest

The authors declare no conflicts of interest.

## Data Availability

All data generated or analyzed during this study are included in this published article.
